# Practical identifiability analysis of a mechanistic model for the time to distant metastatic relapse and its application to renal cell carcinoma

**DOI:** 10.1371/journal.pcbi.1010444

**Published:** 2022-08-25

**Authors:** Arturo Álvarez-Arenas, Wilfried Souleyreau, Andrea Emanuelli, Lindsay S. Cooley, Jean-Christophe Bernhard, Andreas Bikfalvi, Sebastien Benzekry

**Affiliations:** 1 MONC, Mathematical Modeling for Oncology, Inria Bordeaux Sud-Ouest, Talence, France; 2 University of Bordeaux, LAMC, Pessac, France; 3 Inserm U1029, Pessac, France; 4 Urology department, Centre Hospitalier Universitaire (CHU) de Bordeaux, France; 5 COMPO, COMPutational pharmacology and clinical Oncology, Centre Inria Sophia Antipolis - Méditerranée, Centre de Recherches en Cancérologie de Marseille, Inserm U1068, CNRS UMR7258, Institut Paoli-Calmettes, Aix-Marseille University; Dartmouth College, UNITED STATES

## Abstract

Distant metastasis-free survival (DMFS) curves are widely used in oncology. They are classically analyzed using the Kaplan-Meier estimator or agnostic statistical models from survival analysis. Here we report on a method to extract more information from DMFS curves using a mathematical model of primary tumor growth and metastatic dissemination. The model depends on two parameters, *α* and *μ*, respectively quantifying tumor growth and dissemination. We assumed these to be lognormally distributed in a patient population. We propose a method for identification of the parameters of these distributions based on least-squares minimization between the data and the simulated survival curve. We studied the practical identifiability of these parameters and found that including the percentage of patients with metastasis at diagnosis was critical to ensure robust estimation. We also studied the impact and identifiability of covariates and their coefficients in *α* and *μ*, either categorical or continuous, including various functional forms for the latter (threshold, linear or a combination of both). We found that both the functional form and the coefficients could be determined from DMFS curves. We then applied our model to a clinical dataset of metastatic relapse from kidney cancer with individual data of 105 patients. We show that the model was able to describe the data and illustrate our method to disentangle the impact of three covariates on DMFS: a categorical one (Führman grade) and two continuous ones (gene expressions of the macrophage mannose receptor 1 (MMR) and the G Protein-Coupled Receptor Class C Group 5 Member A (GPRC5a) gene). We found that all had an influence in metastasis dissemination (*μ*), but not on growth (*α*).

## Author summary

Understanding biological mechanisms leading to metastasis development is a major challenge in order to prevent distant relapse of cancer. Classical methods to study associations of biomarkers with subsequent metastatic relapse rely on the analysis of metastasis free survival curves by means of statistical models such as proportional hazards Cox regression. These models act as black boxes and don’t provide detailed information about the specific mechanism involved. In our study, we propose to use a method based on mechanistic modeling of the metastatic development, that is, a mathematical model that simulates the biological process. The main challenge for these models is to implement the right level of complexity, because if too many parameters are included, these cannot be precisely identified from the data. We reduced the metastatic process to two main aspects: growth and dissemination. We then proposed a theoretical study of the identifiability of the two associated parameters from metastasis-free survival curves. Eventually, we applied our method to a clinical dataset in kidney cancer and illustrated how we could gain biological insights about the role of some diagnosis markers.

## Introduction

Classical statistical methods for survival analysis (i.e., analysis of right-censored, time-to-event data) comprise Kaplan-Meier estimator, parametric models (based on a specific distribution) and semi-parametric proportional hazard Cox regression (which analyzes the hazard ratio between two groups of patients with different characteristics) [1]. The Kaplan-Meier estimator is used in oncology to analyze time to progression, to metastatic relapse or to death, and can be used to compare two or more groups of subjects [2]. Statistical differences between the curves are usually compared with the log-rank or Breslow test [1]. To analyze the association of covariates with survival, proportional hazard Cox regression modeling is ubiquitous [3].

With the development of machine learning (ML) algorithms, new tools have been developed. In 2008, Ishwaran et al. proposed an extension of the classical random forest algorithm to survival data that uses a splitting rule based on a log-rank test [4]. The Least Absolute Shrinkage and Selection Operator (LASSO) and elastic net ML algorithms have also been extended to Cox regression [5]. More recently, artificial neural networks (deep learning) have been adapted to survival regression [6]. However, these techniques often need large amounts of data to be reliable, and lack biological interpretability.

For that reason, mechanistic models including some of the important biological processes of the problem are emerging as an interesting alternative to analyze distant metastasis-free survival (DMFS) curves [7]. By mechanistic model, we mean here a model that simulates the dynamics of a patho-physiological process (here, tumor growth and dissemination). These models can not only be used to select some important covariates but also to get biological clues about the effect of biomarkers and to make individual and population predictions. This novel approach has demonstrated not only similar predictive power compared with classical statistical survival models (e.g. Cox proportional hazard regression) and machine learning algorithms (e.g., random survival forest), but also ability to bring mechanistic insight on the impact of clinical and biological markers on metastatic processes [7]. However, detailed identifiability properties of the parameters have yet to be established in order to understand and quantify how much mechanistic information can be extracted from DMFS curves.

Here, we performed a practical identifiability study and applied our novel approach to prediction of metastatic relapse in renal cell carcinoma (RCC). RCC is the most common type of kidney cancers in adults [8]. When the disease has not spread, initial treatment consists in partial or complete removal of affected kidney(s) and the 5-year survival rate is relatively good (65–90%) [9]. However, 40% of patients with apparently localized disease will relapse [10]. When metastases are present, therapeutic options are limited and the 5-year survival rate dramatically drops to 13% [9]. Although crucial for determining the best therapeutic option, prognostic biomarkers are lacking in clinical practice. Our computational methodology brings new ways to perform biomarker exploratory studies, in a biologically-informed fashion, in contrast to agnostic statistical learning algorithms.

The paper is organized as follows. First, we present our methodology to: 1) mechanistically model the individual time to distant metastatic relapse, including the processes of primary tumor growth and metastatic dissemination, 2) embed this individual model into a population approach (using the framework of statistical mixed-effects models), 3) integrate biomarkers as covariates in either of growth or dissemination and 4) identify population parameters and covariate coefficients from DMFS curves. Then, we illustrate our approach by analyzing a RCC clinical dataset containing clinical and biological markers together with individual DMFS.

## Materials and methods

### Ethics statement

The study was approved by the ethics committee at each participating center and run in agreement with the International Conference on Harmonization of Good Clinical Practice Guideline.

### Mechanistic model of metastatic dissemination and growth

The mechanistic model of the metastatic process has been detailed in [7]. To make our study self-contained, we briefly summarize the main components.

In RCC, there is evidence suggesting that primary tumor growth is consistent with Gompertzian kinetics [11]. For each individual patient *i*, Gompertzian growth is described by the following equation:

$$\begin{array}{c} V_{p}^{i}\left(t\right)=e^{\left(\frac{\alpha^{i}}{\beta^{i}}(1-e^{\left(-\beta^{i}t\right)})\right)}, \end{array}$$ (1)

where $V_{p}^{i}\left(t\right)$ represents the number of cells of the primary tumor, and *α*^*i*^ and *β*^*i*^ are the Gompertzian growth parameters for the individual *i*. Written with formula 1, the parameter *α*^*i*^ corresponds to the specific growth rate (that is, $SGR\left(t\right)=\frac{1}{V}\cdot \frac{dV}{dt}$), when *V* = 1 cell. The parameter *β*^*i*^ expresses the biological fact that *SGR*(*t*) decreases in time [12, 13]. Specifically, it corresponds to the biological hypothesis that *SGR*(*t*) decreases exponentially fast and *β*^*i*^ is such that *SGR*^*i*^(*t*) = *α*^*i*^*e*^−*β*^*i*^*t*^ [14]. To limit the number of parameters for growth and based on biological evidence, the upper limit $K^{i}=e^{\frac{\alpha^{i}}{\beta^{i}}}$ was assumed to be fixed to 10^12^ [15]. Letter *t* refers to *time* from now on.

In addition, metastasis dissemination *d*^*i*^ is assumed to be proportional to the primary tumor size

$$\begin{array}{c} d^{i}\left(V_{p}^{i}\right)=\mu^{i}V_{p}^{i}, \end{array}$$

where *μ*^*i*^ is the per cell per day probability that a cell from the primary tumor disseminates and establishes a distant metastatic colony. Despite the fact that the metastasis process involves stochastic events, we believe that estimation and quantification of intra-individual variance is not achievable from the macroscopic data considered here. Therefore, we neglect this source of randomness and consider the expected total number of metastasis *N*^*i*^ at time *t*, given by:

$$\begin{array}{c} N^{i}\left(t\right)=\int_{0}^{t}d^{i}(V_{p}^{i}\left(s\right))ds=\int_{0}^{t}\mu^{i}V_{p}^{i}\left(s\right)ds. \end{array}$$

### Mechanistic model of the time to relapse

**Fig 1:**
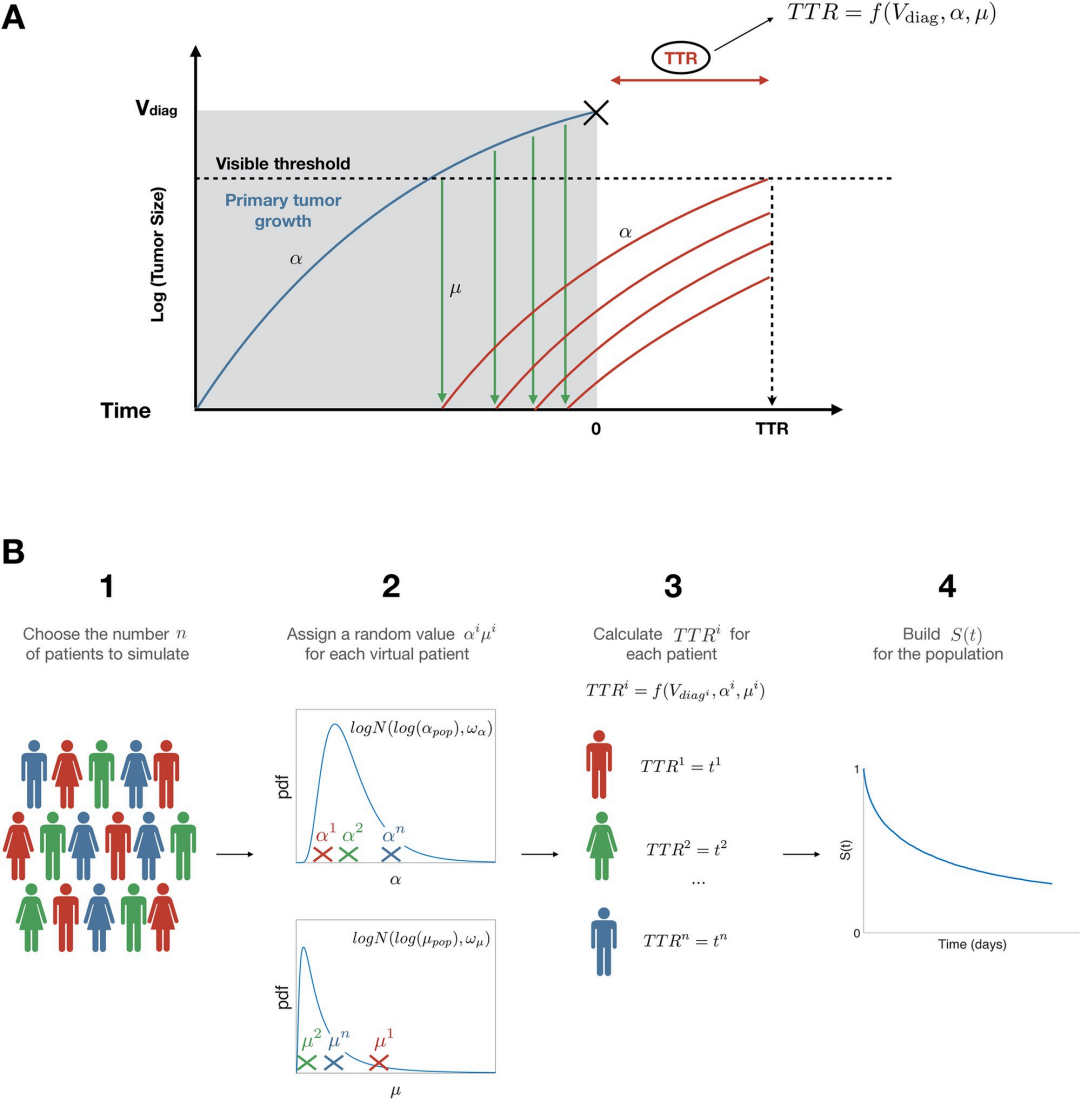
Scheme of the mechanistic model. A) Individual processes (adapted from [7]). Parameter *α* quantifies tumor growth while parameter *μ* quantifies metastatic dissemination. B) Population scheme, with S(t) the survival function of the random variable *TTR*. pdf = probability density function.

The scheme of the model of time to relapse (TTR) can be seen in Fig 1A. Primary tumor and metastasis are assumed to grow at the same rate *α*. This assumption, although debatable, was made to ensure a limited number of parameters, but also based on reported evidence from the literature [16, 17].

We define *τ*_*vis*_ as the time for a tumor to reach the visible threshold *V*_*vis*_ (assumed to be the number of cells corresponding to a diameter of 5 mm under spherical shape assumption and using the conversion 1 mm^3^ = 10^6^ cells [7, 18, 19]). Assuming Gompertzian kinetics, it can be expressed as

$$\begin{array}{c} \tau_{vis}=-\frac{1}{\beta^{i}}\;\text{log}\;(1-\frac{\beta^{i}}{\alpha^{i}}\;\text{log}\left(V_{vis}^{i}\right)). \end{array}$$

Similarly, if we define *t*_*diag*_ as the time between the first cancer cell and the diagnosis of the primary tumor, it can be expressed as

$$\begin{array}{c} t_{diag}^{i}=-\frac{1}{\beta^{i}}\;\text{log}\;(1-\frac{\beta^{i}}{\alpha^{i}}\;\text{log}\left(V_{diag}^{i}\right)), \end{array}$$

where $V_{diag}^{i}$ is the volume of the primary tumor at diagnosis. Defining further the number of visible metastasis $N_{vis}^{i}\left(t\right)=N^{i}\left(t-\tau_{vis}^{i}\right)$, then the theoretical individual *TTR*^*i*^ can be defined as:

$$\begin{array}{c} TTR^{i}\left(V_{diag}^{i};\alpha^{i},\mu^{i}\right)=\{\begin{array}{cc} \underset{t>0}{\text{inf}}\;\{N_{vis}^{i}(t_{diag}^{i}+t;\alpha^{i},\mu^{i})\geq 1\} & \text{if}\;N^{i}\left(t_{diag}^{i};\alpha^{i},\mu^{i}\right)\geq 1,\\ +\infty & \text{if}\;N^{i}\left(t_{diag}^{i};\alpha^{i},\mu^{i}\right)<1. \end{array} \end{array}$$

The *TTR*^*i*^ is therefore a function of $V_{diag}^{i}$ and two individual parameters *α*^*i*^ and *μ*^*i*^. Using a population approach, we further assume that the individual patient parameters are distributed log-normally. Specifically,

$$\begin{array}{c} \text{log}\left(\alpha^{i}\right)=\text{log}\left(\alpha_{pop}\right)+\eta_{\alpha }^{i},\;\text{where}\;\eta_{\alpha }^{i}∼N\left(0,\omega_{\alpha }^{2}\right) \end{array}$$

$$\begin{array}{c} \text{log}\left(\mu^{i}\right)=\text{log}\left(\mu_{pop}\right)+\eta_{\mu }^{i}\;\text{where}\;\eta_{\mu }^{i}∼N\left(0,\omega_{\mu }^{2}\right) \end{array}$$

Then, individual parameters *α*^*i*^ and *μ*^*i*^ are independent and identically distributed random variables, with fixed (population) effect (*α*_*pop*_ or *μ*_*pop*_) and random (individual) effect ($\eta_{\alpha }^{i}$ or $\eta_{\mu }^{i}$). The TTR is therefore a random variable (with respect to distribution in the population), which allows to define the model survival function.

$$\begin{array}{c} S\left(t\right)=P[TTR>t;V_{diag},\alpha_{pop},\mu_{pop},\omega_{\alpha },\omega_{\mu }]. \end{array}$$ (2)

### Covariates

Within our mechanistic framework, we can embed the impact of covariates, either categorical or discrete. The impact of a categorical covariate with *k* levels in tumor growth (*α*) can be simulated as follows:

$$\begin{array}{ccc} \text{log}(\alpha^{i}) & = & \text{log}\left(\alpha_{pop}\right)+\eta_{\alpha }^{i}\;\;\;\;\;\;\text{for}\;\text{the}\;\text{reference}\;\text{level},\\ \text{log}(\alpha^{i}) & = & \text{log}\left(\alpha_{pop}\right)+b_{k}\left|\text{log}\left(\alpha_{pop}\right)\right|+\eta_{\alpha }^{i}\;\text{for}\;\text{level}\;k,\;k>1 \end{array}$$ (3)

where $\eta_{\alpha }^{i}∼N\left(0,\omega_{\alpha }^{2}\right)$ and *b*_*k*_ quantifies the relative impact of level *k* on *α*. A covariate on *μ*^*i*^ can be simulated analogously. For a continuous covariate:

$$\begin{array}{c} \text{log}\left(\alpha^{i}\right)=\text{log}\left(\alpha_{pop}\right)+f\left(x^{i}\right)+\eta_{\alpha }^{i}, \end{array}$$

where *x*^*i*^ is the value of the covariate *x* in patient *i* and *f*(*x*^*i*^) determines the functional relationship between the covariate and the parameter (here, *α*^*i*^). Here, we considered three possible forms:

Threshold effect:

$$\text{log}(\alpha^{i})=\text{log}\left(\alpha_{pop}\right)+\eta_{\alpha }^{i},\;\;\text{if}\;x^{i}\leq c$$ (4)

$$\text{log}(\alpha^{i})=\text{log}\left(\alpha_{pop}\right)+b\left|\text{log}\left(\alpha_{pop}\right)\right|+\eta_{\alpha }^{i},\;\;\;\text{if}\;x^{i}>c$$ (5)

with *b* quantifying the (relative) impact of the covariate and *c* a threshold.

Linear effect:

$$\begin{array}{c} \text{log}\left(\alpha^{i}\right)=\text{log}\left(\alpha_{pop}\right)+b\left|\text{log}\left(\alpha_{pop}\right)\right|x^{i}+c+\eta_{\alpha }^{i}, \end{array}$$ (6)

Combined threshold and linear effect:

$$\text{log}(\alpha^{i})=\text{log}\left(\alpha_{pop}\right)+\eta_{\alpha }^{i},\;\;\text{if}\;x^{i}\leq c$$ (7)

$$\text{log}(\alpha^{i})=\text{log}\left(\alpha_{pop}\right)+b\left|\text{log}\left(\alpha_{pop}\right)\right|x^{i}+\eta_{\alpha }^{i},\;\;\;\text{if}\;x^{i}>c$$ (8)

Similar expressions were considered for an impact on *μ*.

### Parameter estimation and identifiability

#### Objective functions

To estimate the parameter values, we used nonlinear least-square regression applied to the survival curves from the synthetic data sets, using the Matlab function *fminsearch* for minimization (Nelder-Mead algorithm, Matlab2018b) [20]. This algorithm searches for the combination of parameters $\hat{\Theta }=(\hat{\theta_{1}},...,\hat{\theta_{h})}$ that minimizes a specific objective function and has been preferred over other algorithms (e.g., *fmincon*) because it is less prone to converge to local minima, as it is not a gradient-based method. As the algorithm requires an initial condition which might influence the estimation, for each dataset the initial values were randomly chosen using latin hypercube sampling around the real values [21]. We minimized the sum of squared differences between the data *S*_*j*_—either given by the proportion of simulated patients who had not relapsed at time *t*_*j*_ in the synthetic data case, or the Kaplan-Meier estimate for the clinical data—and the model solution (*S*(*t*_*j*_, Θ)). Given that, on one hand, the Kaplan-Meier estimator provides an estimate of the actual survival curve, and on the other hand the model directly simulates uncensored survival, using this method allowed to avoid dealing with censoring, as would be required for maximum likelihood estimation [7]. We considered two possible objective functions. The expression for the first estimator is:

$$\begin{array}{c} \hat{\Theta }=\text{arg}\underset{\Theta }{\text{min}}\sum_{j=1}^{n}{\left(S_{j}-S\left(t_{j},\Theta \right)\right)}^{2}. \end{array}$$ (9)

Given our definition of S(t) (2), S(t = 0) = 1. However, in the data a non-negligible proportion *M* of patients had metastases at diagnosis. Our TTR model also allows for metastasis at diagnosis, in the case $N_{vis}^{i}\left(t_{diag}^{i};\alpha^{i},\mu^{i}\right)>0$. We thus denoted by *m*_*diag*_(Θ) the resulting model-based proportion of patients with metastasis at diagnosis. To account for these considerations, we considered another objective function, defined by:

$$\begin{array}{c} \hat{\Theta }=\text{arg}\underset{\Theta }{\text{min}}\sum_{j=1}^{n}{\left(S_{j}-S\left(t_{j},\Theta \right)\right)}^{2}+{\left(\left(M-m_{diag}\left(\Theta \right)\right)*\lambda \right)}^{2}, \end{array}$$ (10)

where *M* and *m*_*diag*_(Θ) are the fraction of patients with metastasis at diagnosis for the data and model, respectively. The parameter λ balances the two parts of the objective function and was taken to be 0.01 following initial manual explorations.

For coefficients of a categorical covariate, we proceeded similarly and summed the objective functions within each covariate level. For a continuous covariate, survival information was calculated at different thresholds of the covariate (thresholds varying from index *l* = 1, …, *L*). At each threshold, patients were divided into two groups. Group *g* = 1 for those patients with an individual value of the covariate below the threshold, group *g* = 2 for the other. The function that was minimized (in the case of objective function (9)) reads:

$$\begin{array}{c} \left(\hat{b},\hat{c}\right)=\text{arg}\underset{b,c}{\text{min}}\sum_{l=1}^{L}\sum_{g=1}^{2}(\sum_{j=1}^{n}{\left(S_{lgj}-S_{lg}\left(t_{j};b,c\right)\right)}^{2}{)}^{1/2}, \end{array}$$ (11)

where *S*_*lgj*_ is the survival data (at threshold *l*, for the group *g* at time *t*_*j*_). The expression for objective function (10) was similar.

#### Methodology for assessing practical identifiability from simulated data

We simulated synthetic data using the following paramter values: *α*_*pop*_ = 0.005 day^−1^, *μ*_*pop*_ = 7 ⋅ 10^−12^ cell^−1^ day^−1^, *ω*_*α*_ = 1 day^−1^, *ω*_*α*_ = 2.2 cell^−1^ day^−1^. These values were selected to be in the range of clinical values of RCC and previous work [7, 22, 23]. Each synthetic dataset was composed of 1000 patients. To analyze parameter estimation we simulated 200 datasets.

We explored parameter identifiability in multiple possible situations, fixing some parameter values and estimating the others. The step-by-step approach to identify the parameter values was as follows (*K* = 200):

- Using the mechanistic model, we simulated *K* survival dataset of 1000 patients each, generating thus *K* synthetic survival functions *S*_*k*_ = (*S*_*k*1_, …, *S*_*kn*_), with *k* = 1, …, *K*
- We chose an initial condition $\Theta_{0}^{k}$ using latin hypercube sampling
- We estimated parameters values ${\hat{\Theta }}^{k}$ using nonlinear least-square minimization. This resulted in *K* estimated parameters sets.
- Using the *K* estimated parameters sets, we characterized the distribution and confidence intervals of each parameter.

To quantify parametric uncertainty, we calculated the relative standard error (RSE) of each parameter, defined by $RSE=100\cdot \frac{{\left(\frac{1}{K}\sum_{k=1}^{K}{\left(\theta^{*}-{\hat{\theta }}^{k}\right)}^{2}\right)}^{\frac{1}{2}}}{\theta^{*}}$, where θ* represents the true parameter value and ${\hat{\theta }}^{k}$ the estimated parameter in the iteration *k*. Practical identifiability was considered acceptable when RSE were lower than 30% for fixed and random effects.

### Clinical data

#### Parameter values from the literature

The volume at diagnosis was estimated using data from [23]. In this study, pathologically primary tumor volume was measured assuming elliptical shape $PTV=\frac{\pi }{6}\cdot height\cdot length\cdot width$ in 482 patients with RCC. They divided the patients into four groups according to primary tumor size and they provide the information about the mean primary tumor volume and the standard deviation. Assuming normal distribution of the primary tumor volume in each subgroup, we simulated 482 patients according to the frequencies in each subgroup and analyzed the general distribution with the distribution fitter app implemented in Matlab2018b. Data was log-normally distributed with mean 3.196 cm^3^ (*RSE* = 0.0244) and standard deviation 1.711 cm^3^ (*RSE* = 0.0321), where *RSE* is the ratio between the mean standard error (provided by the fitter app) and the estimated value expressed as a percentage.

The parameters involving primary tumor growth were estimated with the information provided in [22]. In that paper, Gofrit et al. provided the distribution information about initial diameter (*d*_0_), time to diagnosis (*t*) and primary tumor growth parameters (*α*_*l*_, which were estimated in *cm*/*year* assuming linear growth). To calculate the values of *α*_*pop*_ and *ω*_*α*_ in our model, we simulated 10000 patients with a random $d_{0}^{i},t^{i}$ and $\alpha_{l}^{i}$ within their distributions and calculated the value of *α*^*i*^ with the following formula.

$$\begin{array}{c} \alpha^{i}=-\frac{\text{log}(10^{12})}{t}\;\text{log}\;(1-3\frac{\text{log}\;(\frac{d_{0}^{i}+\alpha_{l}^{i}\cdot t^{i}}{d_{0}^{i}})}{\text{log}\;(10^{12})}) \end{array}$$

With the individual values of *α*_*i*_ we characterized the population and analyzed the general distribution with the distribution fitter app implemented in Matlab2018b. Growth parameters were log-normally distributed with mean log(*α*_*pop*_) = −3.521 (*RSE* = 0.0031) and standard deviation *ω*_*α*_ = 0.827 (*RSE* = 0.0093).

#### Individual data

Patient samples (primary tumor tissue and plasma) from the UroCCR cohort were used with associated clinical data (clinicaltrial.gov, NCT03293563). Data from 144 patients with RCC was collected between 2006 and 2010. All patients had undergone surgery of the primary tumor, and information about the time of metastatic relapse or alternatively the time of right censoring was available. Metastasis were present mainly in lungs but also in other locations such as bones, lymph nodes, pleura, brain or abdomen. Among all patients, 108 patients had information of at least three biomarkers from tissues samples. For those 108 patients, missing information was completed using the missForest algorithm implemented in R. We focused on three biomarkers, one categorical from histology (Führman grade) and two continuous from quantitative polymerase chain reaction (qPCR) quantification of gene expression from tumor tissues (Macrophage Manose Receptor (MMR) and the G Protein-Coupled Receptor Class C Group 5 Member A (GPRC5a) gene). The continuous covariates were normalized between 0 and 1. The patient data are derived from a national renal cell cancer cohort (UROCCR) which is localized at the University Hospital in Bordeaux France. The study was approved by the ethics committee at each participating center and run in agreement with the International Conference on Harmonization of Good Clinical Practice Guideline. To access the uROCCR database a request should be addressed to the UROCCR Network and the CHU of Bordeaux https://ssl3.isped.u-bordeaux2.fr/UROCCR/Public/Index.aspx. The microarray gene expression data is available via Gene Expression Omnibus using the accession GSE142109.

### Statistical survival analysis

All comparisons made to analyze two or more DMFS curves were done using the log-rank test. When not mentioned otherwise, the significant level was *α* = 0.05. The corresponding null hypothesis was H0: there is no difference between the populations in the probability of an event (here a distant metastatic relapse), at any time point.

## Results

### Identifiability with no covariates

**Fig 2:**
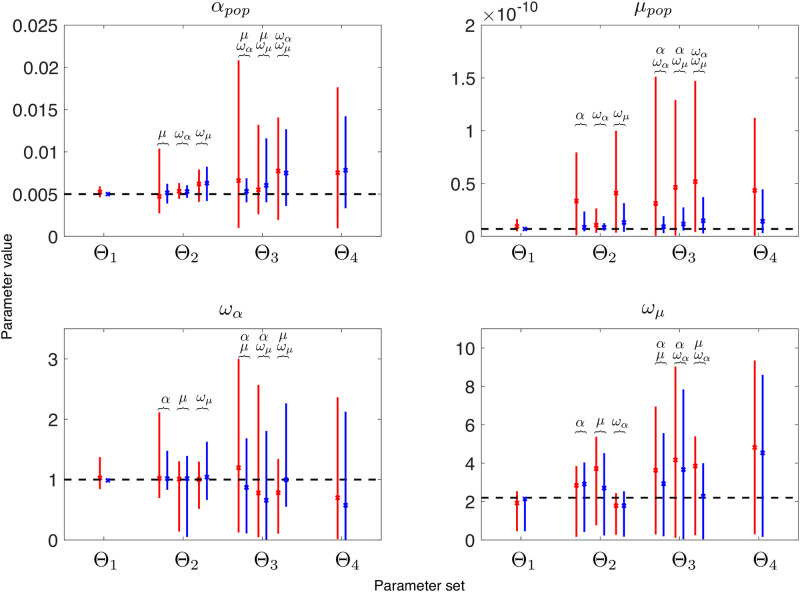
Mean and 95% confidence interval of each estimated parameter. The index *h* in Θ_*h*_ refers to the number of parameters that were jointly estimated. Red and blue lines are the estimations with the first and second objective functions respectively, corresponding to accounting for the initial proportion of metastatic patients (blue) or not (red). The dashed black lines corresponds to the true value of the parameter. The parameters that have also been estimated in each situation are displayed above the solid lines.

We first analyzed the identifiability of the model without covariates. We simulated *K* = 200 datasets of 1000 patients each with the model and estimated the parameters as explained above. As can be observed in Fig 2 and S1 Fig, practical identifiability was good (small RSEs) when only one parameter had to be estimated and worsened when increasing the number of parameters to estimate. Comparing the results between the two objective functions (Eqs 9 vs 10, see red and blue lines in Fig 2), we found that practical identifiability improved when we included also the fraction of patients with metastasis at diagnosis in the objective function. While the RSE and confidence intervals may look similar for the parameters *α*_*pop*_, *ω*_*α*_ and *ω*_*μ*_, the case of *μ*_*pop*_ was different. Identifiability of this parameter improved with the second objective function, with an important decrease in both the RSE and width of the confidence interval. However, with a RSE threshold at 50% for fixed and random effects, not many parameters can be jointly estimated. For the first objective function, the maximum number of parameters that can be estimated together is two, and not in all possible combinations. Parameter *μ* presents high RSE, and can only be estimated alone or in combination with *ω*_*α*_. For the second objective function, the situation is better. Two parameters can always be estimated and there are some combinations in which it is also possible to estimate three parameters at the same time, (*α*_*pop*_, *μ*_*pop*_, *ω*_*α*_) and (*μ*_*pop*_, *ω*_*α*_, *ω*_*μ*_).

### Identifiability with covariates

#### Categorical covariate

**Fig 3:**
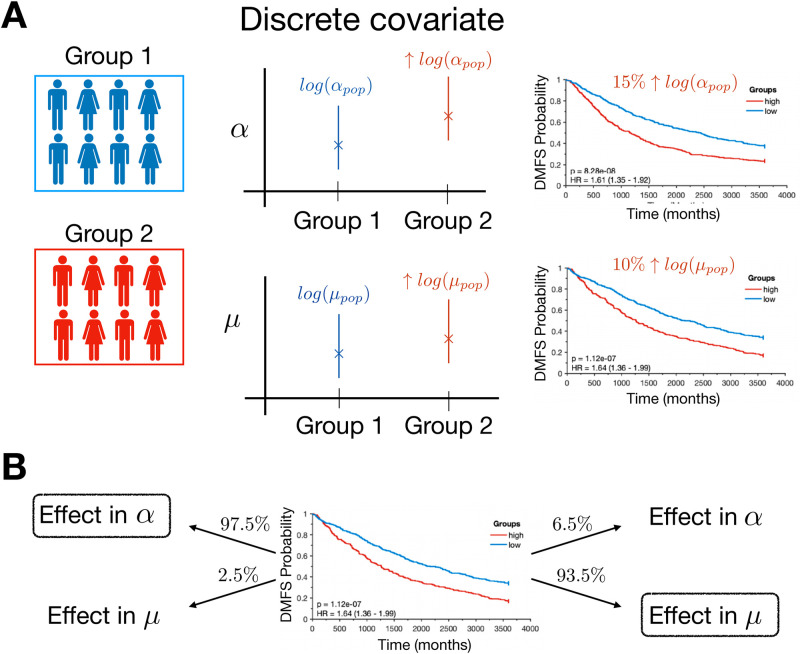
Effect of a categorical covariate. A) Scheme of model simulation with an effect of a categorical covariate in *α* or *μ*. The individual values *α*^*i*^ and *μ*^*i*^ are sampled from different distributions depending on the group. In the right panel, differences in the survival curves are displayed for the two groups. B) Inference of the right effect from the data.

We performed simulations with the model including a categorical covariate. Each simulated patient was randomly assigned into the first or the second group (Bernoulli distribution, p = 1/2). We analyzed survival curves with an effect in *α* and *μ*, and with different values of *b* (see S2(A) and S2(B) Fig). To have statistically significant difference between the two groups (with 1000 patients), the difference in log(*α*_*pop*_) between the two groups had to be around 15%, this being percentage similar but slightly smaller, around 10%, for log(*μ*_*pop*_), see Fig 3A and S2(C) and S2(D) Fig.

In addition, we performed identifiability analysis of the parameter *b* (the other parameter values being fixed). We simulated 200 datasets of 1000 patients. We analyzed RSE and 95% confidence intervals for the mean. The initial condition *b*_0_ was taken close to the real one ($b=0.3,\;b_{0}∼U\left(0.2,0.4\right)$). The RSE of the parameter *b* was below 1%, for effect in *α* (RSE 0.16%, CI (0.25–0.33), b* = 0.3) and in *μ* (RSE 0.41%, CI (0.21–0.41), b* = 0.3).

We also analyzed whether we could detect if the effect was present in *α*_*pop*_ or in *μ*_*pop*_. To that aim, we simulated data with impact in only one parameter (for example, in *α*_*pop*_) and estimated *b* using nonlinear least squares regression for the effect in *α*_*pop*_ or in *μ*_*pop*_. Then, we compared the minimum value of each objective function. In 97.5% of the cases, when the effect of the covariate was in *α*_*pop*_ the residual in nonlinear least squares regression was lower for *α* than for *μ*. Moreover, in 93.5% of the cases the residual was lower for *μ* when the effect was in *μ*_*pop*_, see Fig 3B.

#### Continuous covariate

**Fig 4:**
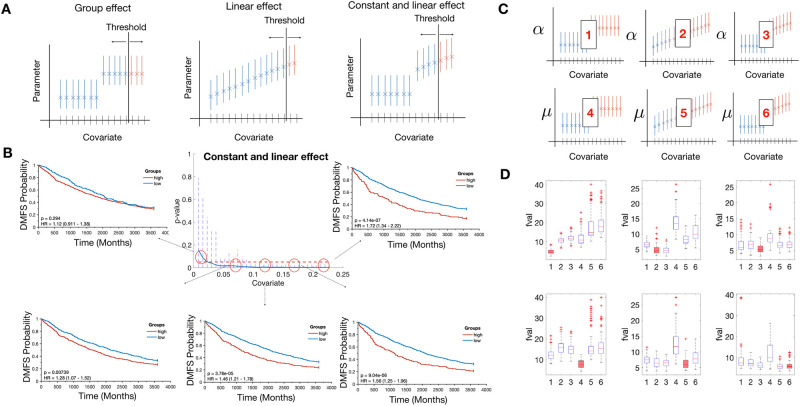
Effect of a continuous covariate. A) Individual values of *α*^*i*^ or *μ*^*i*^ are taken from different distributions depending on the covariate value and the type of effect. B) Synthetic DMFS curves at different thresholds simulated with a constant and linear effect in the variable *α*. P-values at different thresholds are displayed in the center figure C) Scheme of inferring the right effect from the data. Number 1–3 refers to effects group, linear, constant and linear in the variable *α*_*pop*_ and 4–6 in the variable *μ*_*pop*_. D) Results from the minimization process with a gamma distribution for the covariate. Red box plots correspond to the real model used to generate the data with the model and *Fval* is the value of the objective function with the parameter estimated.

We also performed simulations in the case of a continuous covariate. We simulated three qualitatively different possible effects in *α* and *μ* (threshold, linear and threshold then linear, see Fig 4A). To simulate a continuous variable we assigned a random value *x*^*i*^ for each virtual patient. To analyze the possible effect of the covariate distribution on the survival curves we simulated three different covariate distributions. To analyze the effect in comparison with the population values of *α* and *μ*, these three distributions were sampled between 0 and 1. The three distributions were a normal distribution (N(0.5,0.1)), a gamma distribution (Γ(0.5, 0.3)) and a log-normal distribution (LN(-2,0.6)), all truncated and renormalized to stand between 0 and 1. Afterwards, we assigned the individual parameter *α*^*i*^ or *μ*^*i*^ with Eqs 4, 5, 6 or 7 and 8 depending on the effect. Once the individual TTR were calculated, we divided the population into two groups (group 1 if *x*^*i*^ < *Th*, group 2 if *x*^*i*^ > *Th*, *Th* being a threshold value for the covariate) and calculated the differences in the survival curves between the two groups.

The different effects could provide similar survival curves when the population was separated into two groups by the threshold which produced the best separation possible. However, when we analyzed the difference between the two groups at several thresholds (from the 15^*th*^ to the 85^*th*^ percentile), the different effects resulted in different behaviors. For example, when we simulated 1000 patients with the threshold effect in *μ* with *b* = 0.1, *c* = 0.5, in percentiles close to the 15^*th*^ the differences between curves were not statistically significant. The differences between the groups became larger as the threshold was closer to *c*, and became smaller again as the threshold moved away from *c*, see S3(A) Fig. With a linear effect in *α* (*b* = 0.5, *c* = 0) and a gamma distribution for the covariate, the differences between the two groups were similar for all the thresholds. Whatever the initial percentiles, the difference in the curves was similar as well as the p-values (close to 10^−9^), see S3(B) Fig. In addition, a constant and linear effect in *α* (*b* = 0.5, *c* = 0.05, gamma distribution for the covariate) provided also a different scenario. In the initial percentiles, there was no statistically significant difference between the groups. The difference between the groups was continuously amplified when the threshold was shifted to higher values of the covariate, being the highest difference achieved at the last threshold Fig 4B.

We also performed an identifiability analysis for the covariate parameters in all situations. In all cases, we found good parametric identifiability, with values RSE values below 5% (Table 1). The minimization process was repeated 100 times for each situation. The different effects were simulated in the variables *α* and *μ*. The functional form and covariate distribution had a minor impact in the identifiability of the parameters.

**Table 1:** RSE for the parameters *b* and *c* True values were b* = 0.3 for group effect, b* = 0.7 for linear and constant and linear and c* = 0.5 for normal distribution, c* = 0.05 for gamma distribution and c* = 0.13 for log-normal distribution, except for linear effect, in which c* = 0.1 independently of the distribution.

		Group			Linear			Constant and linear		
		N	Γ	LN	N	Γ	LN	N	Γ	LN
*α*_*pop*_	b	0.19	0.28	0.21	0.15	1.53	1.36	0.4	2.13	1.86
	c	0.02	0.16	0.03	3.55	4.87	4.05	0.25	3.03	0.68
*μ*_*pop*_	b	2.92	1.17	0.9	0.94	2.13	1.05	3.75	2.5	1.61
	c	0.98	0.17	0.08	1.19	4.86	1.04	2.01	0.8	0.52

In addition, we performed simulations to analyze whether we could infer in what variable and with what functional form a covariate was impacting (scheme in Fig 4C). We created 100 datasets of 2000 patients simulating one type of effect in one variable for a given covariate distribution. Afterwards, we performed nonlinear least-square regression with all the possible effects in either *α* or *μ*. Results of the minimization process are reported in Fig 4D and S4 Fig, panels AB. In most of the cases, the effect and the variable were well recognized from the data (e.g., group effect in *α* with gamma distribution for the covariate). In other cases however, the values of the objective function were very similar among the cases. Importantly, the correct effect and variable were always among the lowest values, therefore never a wrong variable or effect was clearly suggested as the right one from the data and the analysis.

### Application to metastatic relapse in renal cell carcinoma

**Fig 5:**
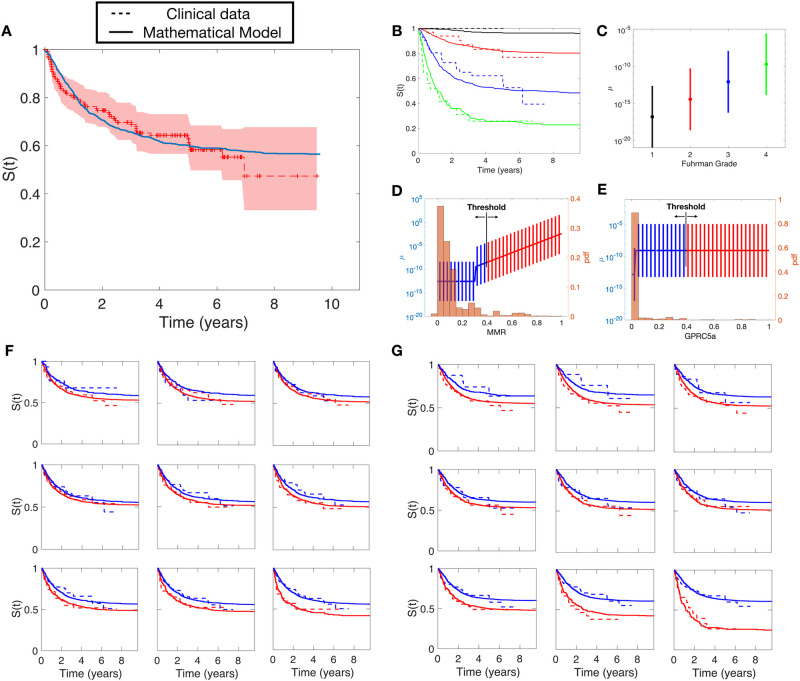
Results of the mathematical model applied to the clinical dataset. A) Goodness-of-fit between the model without covariates and the Kaplan-Meier estimator of the clinical data. B) Goodness-of-fit between the model with the effect of FG in *μ* and the data separated by FG groups. Individual *μ*_*i*_ distributions according to values of C) Führman Grade, D) MMR, E) GPRC5a F) Goodness-of-fit for the model with a constant and linear effect in *μ* for the covariate MMR. The different subfigures are the fits obtained for different thresholds. Dashed lines correspond to the clinical data and solid lines correspond to simulations. Blue lines are the results for group 1 (*MMR*_*i*_ < *threshold*) and red lines for group 2 (*MMR*_*i*_ ≥ *threshold*). G) Goodness-of-fit of the fit between the model with a group effect in *μ* for the covariate GPRC5a.

In this section, we applied the model to a real case. To improve identifiability based on our results above, the values of *α*_*pop*_ and *ω*_*α*_ were estimated from the literature (see Methods). The remaining values were estimated using nonlinear least-square regression with the objective function (10), where *M* = 16, which is the percentage of patients of kidney cancer with metastatic disease at diagnosis [24]. The results of the fit can be seen in Fig 5A.

We analyzed the effect of the categorical covariate Führman Grade (FG) with our model. To do that, we minimized the squared differences between the different groups (we excluded the group FG 1 due to the presence of only one patient having this value in the clinical dataset with this value). For each virtual patient, we assigned a random FG value resampling from the FG distribution of the clinical dataset. Nonlinear least-square regression was performed 50 times using different initial conditions for the parameters *b*_*k*_ (*k* = 3, 4) in Eq 4. 2. The sum of squared differences between the clinical data and the model with effect in *μ* was fval = 0.997 while the sum of squared differences between the clinical data and the model with effect in *α* was fval = 1.861. Therefore, this analysis suggested that FG has an effect in *μ*. In addition, we found that a minimal model with *b*_*k*_ = *b* ⋅ *k* was able to describe the data accurately (Fig 5B). Resulting distributions of parameter *μ* in each FG group are plotted in Fig 5C.

We also analyzed the effect of the continuous covariate MMR in the DMFS curve. The data was analyzed using 15 different thresholds (from percentile 15 to 85, steps of 5) creating two groups for each threshold. We performed nonlinear least-square regression with objective function given by Eq 11 in all possible situations (all effects in *α* and *μ*). Among all of them, the best fits were achieved in the model with a constant and linear effect in *μ* (fval = 9.19) and group effect (fval = 9.26). The rest of the minimization function values were 10.36 (group effect in *α*), 10.49 (linear effect in *α*), 10.46 (constant and linear effect in *α*) and 10.25 (linear effect in *μ*). Distributions of parameter *μ* in each value of the covariate are plotted in Fig 5D (Fig 5E for GPRC5a) and the results of the fits can be seen in Fig 5F. Interestingly, this analysis suggests a nonlinear effect of MMR in the metastatic process. The model provided good agreement with the data in the different thresholds analyzed.

Similarly, we performed the same analysis with the covariate GPRC5a. Results from nonlinear least-square regression suggested that the covariate GPRC5a has an effect in the variable *μ*, with best results for a group effect, *fval* = 9.60. The rest of the values were 14.04 (group effect in *α*), 14.39 (linear effect in *α*), 14.39 (constant and linear effect in *α*), 10.40 (linear effect in *μ*) and 10.41 (constant and linear effect in *μ*). The distributions of parameter *μ*^*i*^ as a function of GPRC5a expression are plotted in Fig 5E and the results of the fits can be seen in Fig 5G.

The values of all the parameters for the model including the covariates are reported in Table 2.

**Table 2:** Estimated parameters values. The parameter *dif*_*μ*,*c*_ has been set to compensate for the unknown value of the parameter *μ*_*pop*_ when studying the covariate *c* (*μ*_*pop*,*c*_ = *μ*_*pop*_ + *dif*_*μ*,*c*_). a.u. = arbitrary unit. RSE = relative standard error.

	value	unit	RSE	estimated from
log(*α*_*pop*_)	-3.521	day^−1^	0.30	[22]
*ω*_*α*_	0.827	day^−1^	0.93	[22]
log(*μ*_*pop*_)	-29.054	cell^−1^ day^−1^	2.10	data
*ω*_*μ*_	4.905	cell^−1^ day^−1^	15.40	data
*b*_*μ*,*FG*_	5.4354	a.u	7.02	data
*dif*_*μ*,*FG*_	-15.1139	cell^−1^ day^−1^	9.09	data
*b*_*μ*,*MMR*_	0.80	a.u	18.27	data
*c*_*μ*,*MMR*_	0.30	a.u	24.10	data
*dif*_*μ*,*MMR*_	0.15	cell^−1^ day^−1^	10.69	data
*b*_*μ*,*GPRC*5*a*_	0.2987	a.u	27.72	data
*c*_*μ*,*GPRC*5*a*_	0.03	a.u	25.50	data
*dif*_*μ*,*GPRC*5*a*_	-0.45	cell^−1^ day^−1^	36.10	data

## Discussion

Classical survival analysis models such as proportional hazard Cox regression are ubiquitous for time-to-event analysis. However, due to their agnostic nature (they only model the survival relapse hazard), they can only lead to a statistical association between covariates (biological markers) and survival and cannot inform on the specific biological process impacted by the biomarker. Conversely, using our mechanistic model, we are able to distinguish between an effect on growth or dissemination. Specifically, in our analysis we found that FG, MMR and GPRC5a have an impact on metastasis dissemination. In previous studies (in breast cancer), we had concluded that other factors (e.g., Ki67), impacted on growth rather than dissemination [7]. In addition, with our approach, we could theoretically make more precise and more complete predictions, such as the amount of minimal residual disease invisible at diagnosis and after surgery, or the specific TTR of a given patient.

In this paper, we used a mechanistic model of tumor growth and metastatic dissemination that had been previously introduced to describe metastatic development [25]. The definition of a mechanistic model (here taken to be a model that simulates a patho-physiological process) is arguable as the model parameters are not directly measurable by biological assays. Other authors might describe such simulation models as “phenomenological” [26]. Our model simplifies the dynamics of tumor growth and metastases formation. Some extensions could incorporate different growth for primary and secondary tumors, a different dissemination formula taking into account that only a small fraction of cancer cells can disseminate (using a more general expression *d* = *μV*^*θ*^ for some *θ* > 0), or the fact that only vascular tumors can metastasize. In addition, other growth laws different than Gompertzian kinetics could also been explored. Another debatable assumption was to assume that the volume of the first metastasis at relapse was the same for all patients. However, this information (size of the metastases at relapse) is usually not reported in registries such as the one we worked with and we were forced to such assumption here, which we believe does not substantially affect the results. This version of the model is useful for simple approaches and has been successfully applied to several cancers, including breast cancer, lung cancer, neuroblastoma and RCC [27–32]. Only recently has this model been applied to integrate DMFS data, for early-stage breast cancer [7].

We studied here the practical identifiability of parameters of the mechanistic model embedded into a mixed-effects statistical framework. Structural identifiability—although important for theoretical analysis [33, 34]—was beyond the scope of our study because our aim was focused on practical applications to clinical data. We found that the uncertainty about the parameter values was important when both *α*_*pop*_ and *μ*_*pop*_ were estimated together. However, practical identifiability improved with a second objective function that included the percentage of patients with metastasis at diagnosis. In such a case, up to three parameters (e.g. *μ*_*pop*_, *α*_*pop*_ and *ω*_*α*_) could be inferred with reasonable confidence from DMFS data.

Nevertheless, the uncertainty of some parameters was still important when the four parameters (*μ*_*pop*_, *α*_*pop*_, *ω*_*μ*_, *ω*_*α*_) were estimated together. Thus, when applying our model to a clinical kidney cancer dataset, we decided to include parameter values derived from the literature. As it is difficult to obtain quantitative data on metastatic dissemination from clinical studies, we focused on data on primary tumor growth. Several studies analyzing RCC growth by comparing two clinical images at two different time points have been reported [11, 35]. However in this case, only small and slow-growing primary tumors were measured, with the primary tumor growth parameters being underestimated. We decided to obtain information from [22], in which growth of “clinically significant” renal cancer, including all types of primary tumors and sizes, had been analyzed. In this study, 46 patients with RCC were included, all of them having a medical image showing no evidence of kidney cancer from 6 to 60 months prior to the diagnosis. The authors assumed that macroscopic primary tumor growth started shortly after normal imaging. This assumption has consequences for the estimation of primary tumor growth. Nevertheless, these primary tumor growth values matched better with our simulations and were therefore included in our analysis.

We also analyzed the effect of categorical and continuous covariates on tumor growth and metastatic dissemination in our TTR model. We defined a general model in which the effect of a continuous covariate can take have three possible functional forms: stepwise, linear or stepwise then linear combined. Results of parametric identifiability performed with synthetic data were good independently of the effect and the covariates distribution, with RSE below 5% in all cases.

One of the novelties of the approach is the analysis of the effect of the covariates on the different processes. First we did not impose a linear dependency of the parameters on the covariates, allowing for more freedom when analyzing complex biological processes, where the assumption of linearity may not be the most suitable. Second, we dichotomized survival curves with several thresholds. This approach has been previously used in [36] to select the threshold that best separates two groups in Kaplan-Meier analysis (lowest p-value using log-rank test). We hypothesized that group separation according to different thresholds could provide information about the type of the covariate effect. To prove it, we generated synthetic data assuming different effects. We included the differences between the model and the synthetic data in several thresholds in the objective function of the non-linear least squares regression and concluded that it was possible to identify the functional type of effect that generated the data in most of the cases.

For illustration of our methodological approach in a clinically meaningful setting, we applied our method to a clinical kidney cancer dataset. For illustrative purposes, we selected three covariates, Führman Grade, MMR and GPRC5a. The model was able to accurately fit the data and reproduce the impact of each covariate. Consistently with its biological definition associated with tumor aggressiveness [37], increased Führman grade was associated with larger *μ* and thus higher metastatic propensity. For MMR, the analysis suggested a threshold then linear functional form in *μ*. This means that the effect of the variable in the metastatic process has less importance for lower values of the covariate, but will become more important after a threshold in which the effect increases with higher values of the covariate. Increased expression of MMR was also associated with larger *μ* (Table 2). This is consistent with the biological interpretation of the mannose receptor (cluster of differentiation 206, CD206) as indicative of type 2, pro-tumor macrophage phenotype [38, 39]. For GPRC5a, the analysis suggested a group effect, i.e. a threshold in the impact of GPRC5a on *μ*. Association was also positive, suggesting that higher levels of GPRC5a expression are associated with increased metastatic dissemination. This association of GPRC5a with metastatic potential corroborates with this gene being an emerging biomarker of human cancer [40]. These biological conclusions should be further confirmed using a large dataset with information about primary tumor volumes. To lead to a clinically applicable model, the predictive abilities should be more thoroughly studied using cross-validation on the current model development set but also testing model predictions in an external data set. In addition, more advanced biological processes might be added to the model (e.g., dormancy [31] or post-surgery metastatic acceleration [41]), and more biomarkers available at diagnosis could be integrated as covariates, including omic data. This last point could lead to non-trivial identifiability issues requiring further methodological developments. Last, a major feature to add would be the integration of (neo-) adjuvant treatment.

In summary, we have analyzed the identifiability properties of our mechanistic approach to study distant metastatic-free survival curves. This allowed to derive biological insights from distant metastatic-free survival curves when classical statistical approaches from survival analysis can only bring correlative information.

## Supporting information

S1 Fig Relative standard errors (RSE) of the general parameters. RSE of each parameter in each situation. The index *i* in Θ_*i*_ refers to the number of parameters that has been jointly estimated. Red and blue bars are the RSE with the first and second objective functions respectively. The parameters that has also been estimated in each situation are displayed above the bars.
(PDF)

S2 Fig Discrete covariate. Survival curves between groups with different levels values of *b*: A) Effect in *α*, B) effect in *μ*, C-D)Solid line represents the mean p-value of log-rank test with different values of *b*. Dashed blue lines represents the 95% CI. Red dashed line represents the statistical significance level *p* = 0.05. Simulations were repeated 100 times. C) Effect in *α* D) Effect in *μ*.
(PDF)

S3 Fig Different effects in a continuous variable. Synthetic DMFS curves at different thresholds simulated with a effect in the variable *α*. P-values at different thresholds are displayed in the center figure. A) Group effect B) Linear effect.
(PDF)

S4 Fig Identification of the correct effect with a continuous covariate. Results from minimization process. Number 1–3 refers to effects group, linear, group and linear in the variable *α*_*pop*_ and 4–6 in the variable *μ*_*pop*_. Red boxplots correspond to the real model used to generate the data with the model. A) Normal distribution for the covariate, B) Lognormal distribution.
(PDF)

## References

[1] Collett D. Modelling survival data in medical research. CRC Press Taylor & Francis Group; 2015.

[2] Kaplan EL, Meier P. Nonparametric Estimation from Incomplete Observations. Journal of the American Statistical Association. 1958;53(282):457–481. doi: 10.1080/01621459.1958.10501452

[3] Cox DR. Regression Models and Life-Tables. Journal of the Royal Statistical Society Series B (Methodological). 1972;34(2):187–220. doi: 10.1111/j.2517-6161.1972.tb00899.x

[4] Ishwaran H, Kogalur UB, Blackstone EH, Lauer MS. Random survival forests. The Annals of Applied Statistics. 2008;2(3):841–860. doi: 10.1214/08-AOAS169

[5] Simon N, Friedman J, Hastie T, Tibshirani R. Regularization Paths for Cox’s Proportional Hazards Model via Coordinate Descent. J Stat Softw. 2011;39(5):1–13. doi: 10.18637/jss.v039.i05 27065756 PMC4824408

[6] Yousefi S, Amrollahi F, Amgad M, Dong C, Lewis JE, Song C, et al. Predicting clinical outcomes from large scale cancer genomic profiles with deep survival models. Scientific Reports. 2017;7(1):11707. doi: 10.1038/s41598-017-11817-6 28916782 PMC5601479

[7] Nicolò C, Périer C, Prague M, Bellera C, MacGrogan G, Saut O, et al. Machine Learning and Mechanistic Modeling for Prediction of Metastatic Relapse in Early-Stage Breast Cancer. JCO Clinical Cancer Informatics. 2020; p. 259–274. 32213092 10.1200/CCI.19.00133

[8] Hsieh JJ, Purdue MP, Signoretti S, Swanton C, Albiges L, Schmidinger M, et al. Renal cell carcinoma. Nature Reviews Disease Primers. 2017;3. doi: 10.1038/nrdp.2017.9 28276433 PMC5936048

[9] Howlader N, Noone A, Krapcho M, Miller D, Brest A, Yu M, et al. SEER Cancer Statistics Review, 1975-2016. National Cancer Institute Bethesda, MD. 2019.

[10] Society AC. Key Statistics about kidney cancer; 2016. http://www.cancer.org/cancer/kidney-cancer.html.

[11] Crispen PL, Viterbo R, Boorjian SA, Greenberg RE, Chen DYT, Uzzo RG. Natural history, growth kinetics, and outcomes of untreated clinically localized renal tumors under active surveillance. Cancer. 2009;115(13):2844–2852. doi: 10.1002/cncr.24338 19402168 PMC2860784

[12] Norton L. A Gompertzian model of human breast cancer growth. Cancer Research. 1988;48(24):7067–7071. 3191483

[13] Benzekry S, Lamont C, Beheshti A, Tracz A, Ebos JML, Hlatky L, et al. Classical mathematical models for description and prediction of experimental tumor growth. PLoS Computational Biology. 2014-08;10(8):e1003800. doi: 10.1371/journal.pcbi.1003800 25167199 PMC4148196

[14] Vaghi C, Rodallec A, Fanciullino R, Ciccolini J, Mochel JP, Mastri M, et al. Population modeling of tumor growth curves and the reduced Gompertz model improve prediction of the age of experimental tumors. PLoS Computational Biology. 2020;16(2):e1007178. doi: 10.1371/journal.pcbi.1007178 32097421 PMC7059968

[15] Coumans FAW, Siesling S, Terstappen LWMM. Detection of cancer before distant metastasis. BMC Cancer. 2013;13(1):283. doi: 10.1186/1471-2407-13-283 23763955 PMC3684526

[16] Steel GG, Lamerton LF. The growth rate of human tumours. British Journal of Cancer. 1966;20(1):74–86. doi: 10.1038/bjc.1966.9 5327764 PMC2008056

[17] Demicheli R. Growth of testicular neoplasm lung metastases: Tumor-specific relation between two Gompertzian parameters. European Journal of Cancer. 1980-12;16(12):1603–1608. doi: 10.1016/0014-2964(80)90034-1 7227433

[18] Kundel HL. Predictive value and threshold detectability of lung tumors. Radiology. 1981;139(1):25–29. doi: 10.1148/radiology.139.1.7208937 7208937

[19] MacMahon H, Austin JHM, Gamsu G, Herold CJ, Jett JR, Naidich DP, et al. Guidelines for Management of Small Pulmonary Nodules Detected on CT Scans: A Statement from the Fleischner Society1. Radiology. 2005;237(2):395–400. doi: 10.1148/radiol.2372041887 16244247

[20] Lagarias JC, Reeds JA, Wright MH, Wright PE. Convergence Properties of the Nelder–Mead Simplex Method in Low Dimensions. SIAM Journal on Optimization. 1998;9:112–147. doi: 10.1137/S1052623496303470

[21] McKay MD, Beckman RJ, Conover WJ. A Comparison of Three Methods for Selecting Values of Input Variables in the Analysis of Output from a Computer Code. Technometrics. 1979;21:239. doi: 10.1080/00401706.1979.10489755

[22] Gofrit ON, Yutkin V, Zorn KC, Duvdevani M, Landau EH, Hidas G, et al. The growth rate of “clinically significant” renal cancer. SpringerPlus. 2015. doi: 10.1186/s40064-015-1385-9 26543715 PMC4628034

[23] Choi SM, Choi DK, Kim TH, Jeong BC, Seo SI, Jeon SS, et al. A Comparison of Radiologic Tumor Volume and Pathologic Tumor Volume in Renal Cell Carcinoma (RCC). Plos One. 2015;10(3). doi: 10.1371/journal.pone.0122019 PMC4370411 25799553

[24] Diaz de Leon A, Pirasteh A, Costa DN, Kapur P, Hammers H, Brugarolas J, et al. Current Challenges in Diagnosis and Assessment of the Response of Locally Advanced and Metastatic Renal Cell Carcinoma. RadioGraphics. 2019;39(4):998–1016. doi: 10.1148/rg.2019180178 31199711 PMC6677287

[25] Iwata K, Kawasaki K, Shigesada N. A Dynamical Model for the Growth and Size Distribution of Multiple Metastatic Tumors. Journal of Theoretical Biology. 2000;203:177–186. doi: 10.1006/jtbi.2000.1075 10704301

[26] Bajzer Z, Pavelić K, Vuk-Pavlović S. Growth self-incitement in murine melanoma B16: a phenomenological model. Science. 1984;225(4665):930–932. doi: 10.1126/science.6382606 6382606

[27] Baratchart E, Benzekry S, Bikfalvi A, Colin T, Cooley LS, Pineau R, et al. Computational Modelling of Metastasis Development in Renal Cell Carcinoma. PLOS Computational Biology. 2015;11:e1004626. doi: 10.1371/journal.pcbi.1004626 26599078 PMC4658171

[28] Benzekry S, André N, Benabdallah A, Ciccolini J, Faivre C, Hubert F, et al. Modeling the Impact of Anticancer Agents on Metastatic Spreading. Mathematical Modelling of Natural Phenomena. 2012;7:306–336. doi: 10.1051/mmnp/20127114

[29] Benzekry S, Sentis C, Coze C, Tessonnier L, André N. Development and Validation of a Prediction Model of Overall Survival in High-Risk Neuroblastoma Using Mechanistic Modeling of Metastasis. JCO Clinical Cancer Informatics. 2021; p. 81–90. doi: 10.1200/CCI.20.00092 33439729

[30] Benzekry S, Tracz A, Mastri M, Corbelli R, Barbolosi D, Ebos JML. Modeling Spontaneous Metastasis following Surgery: An In Vivo-In Silico Approach. Cancer Research. 2015;76:535–547. doi: 10.1158/0008-5472.CAN-15-1389 26511632 PMC5846333

[31] Bilous M, Serdjebi C, Boyer A, Tomasini P, Pouypoudat C, Barbolosi D, et al. Quantitative mathematical modeling of clinical brain metastasis dynamics in non-small cell lung cancer. Scientific Reports. 2019;9. doi: 10.1038/s41598-019-49407-3 31506498 PMC6736889

[32] Serre R, Benzekry S, Padovani L, Meille C, André N, Ciccolini J, et al. Mathematical Modeling of Cancer Immunotherapy and Its Synergy with Radiotherapy. Cancer Research. 2016;76:4931–4940. doi: 10.1158/0008-5472.CAN-15-3567 27302167

[33] Hanin LG. Identification problem for stochastic models with application to carcinogenesis, cancer detection and radiation biology. Discrete Dynamics in Nature and Society. 2002;7(3):177–189. doi: 10.1080/1026022021000001454

[34] Hanin L, Seidel K, Stoevesandt D. A “universal” model of metastatic cancer, its parametric forms and their identification: what can be learned from site-specific volumes of metastases. Journal of Mathematical Biology. 2015;72(6):1633–1662. doi: 10.1007/s00285-015-0928-6 26307099

[35] Lee SW, Sung HH, Jeon HG, Jeong BC, Jeon SS, Lee HM, et al. Size and Volumetric Growth Kinetics of Renal Masses in Patients With Renal Cell Carcinoma. Urology. 2016;90:119–125. doi: 10.1016/j.urology.2015.10.051 26790589

[36] Pérez-Beteta J, Molina-García D, Ortiz-Alhambra JA, Fernández-Romero A, Luque B, Arregui E, et al. Tumor Surface Regularity at MR Imaging Predicts Survival and Response to Surgery in Patients with Glioblastoma. Radiology. 2018;288:171051. doi: 10.1148/radiol.2018171051 29924716

[37] Fuhrman SA, Lasky LC, Limas C. Prognostic significance of morphologic parameters in renal cell carcinoma. The American Journal of Surgical Pathology. 1982;6(7):655–664. doi: 10.1097/00000478-198210000-00007 7180965

[38] Allavena P, Sica A, Solinas G, Porta C, Mantovani A. The inflammatory micro-environment in tumor progression: The role of tumor-associated macrophages. Critical Reviews in Oncology/Hematology. 2008;66(1):1–9. doi: 10.1016/j.critrevonc.2007.07.004 17913510

[39] Jaynes JM, Sable R, Ronzetti M, Bautista W, Knotts Z, Abisoye-Ogunniyan A, et al. Mannose receptor (CD206) activation in tumor-associated macrophages enhances adaptive and innate antitumor immune responses. Science Translational Medicine. 2020;12(530):eaax6337. doi: 10.1126/scitranslmed.aax6337 32051227 PMC7832040

[40] Jiang X, Xu X, Wu M, Guan Z, Su X, Chen S, et al. GPRC5A: An Emerging Biomarker in Human Cancer. BioMed Research International. 2018;2018:1–11. doi: 10.1155/2018/1823726 30417009 PMC6207857

[41] Hanin L, Rose J. Suppression of Metastasis by Primary Tumor and Acceleration of Metastasis Following Primary Tumor Resection: A Natural Law? Bulletin Mathematical Biology. 2018;80(3):519–539. doi: 10.1007/s11538-017-0388-9 29302774

