# Supplementary material for: Practical identifiability analysis of a mechanistic model for the time to distant metastatic relapse and its application to renal cell carcinoma
Source: PLoS Comput Biol. 2022 Aug 25;18(8):e1010444. doi: 10.1371/journal.pcbi.1010444 (PMC9451098; doi:10.1371/journal.pcbi.1010444)
Supplement: S1 Fig — RSE of each parameter in each situation. The index i in Θi refers to the number of parameters that has been jointly estimated. Red and blue bars are the RSE with the first and second objective functions respectively. The parameters that has also been estimated in each situation are displayed above the bars. (PDF) [file pcbi.1010444.s001.pdf]

S1 Fig: RSE of the general parameters

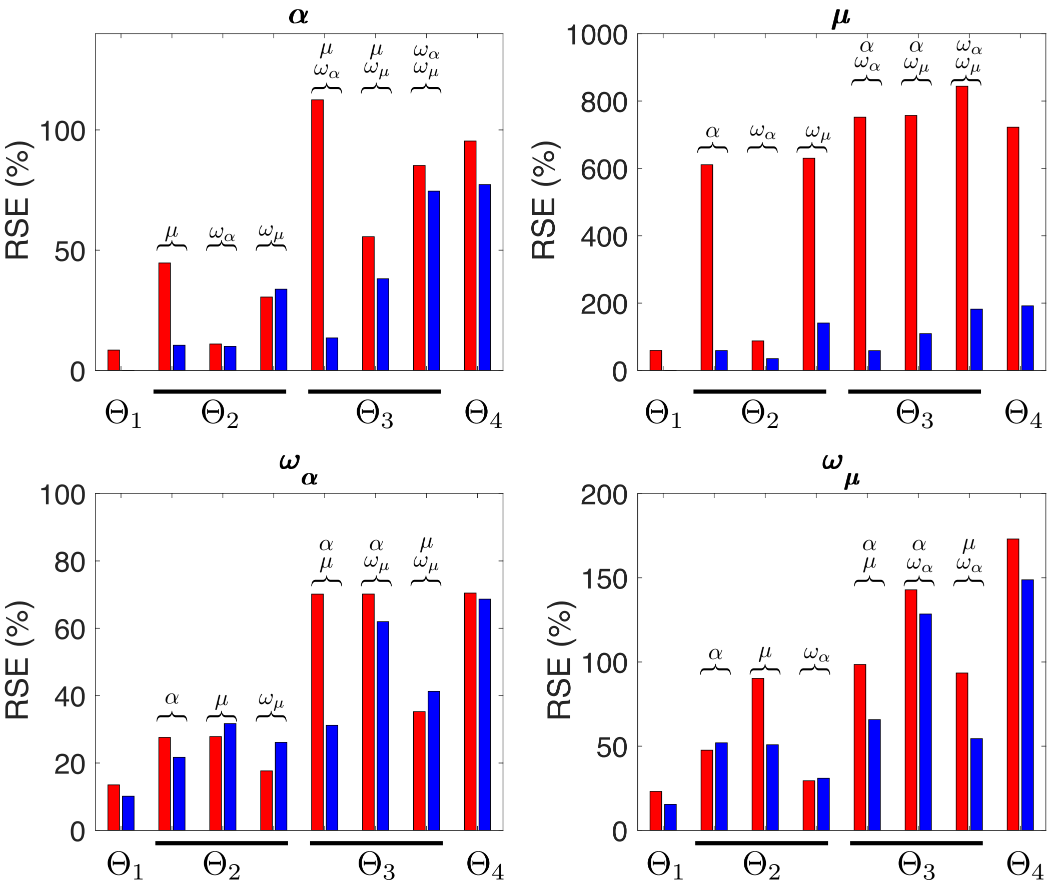

RSE of each parameter in each situation. The index i in Θ_i refers to the number of parameters that has been jointly estimated. Red and blue bars are the RSE with the first and second objective functions respectively. The parameters that has also been estimated in each situation are displayed above the bars.
