# Supplementary material for: Practical identifiability analysis of a mechanistic model for the time to distant metastatic relapse and its application to renal cell carcinoma
Source: PLoS Comput Biol. 2022 Aug 25;18(8):e1010444. doi: 10.1371/journal.pcbi.1010444 (PMC9451098; doi:10.1371/journal.pcbi.1010444)
Supplement: S2 Fig — Survival curves between groups with different levels values of b: A) Effect in α, B) effect in μ, C-D)Solid line represents the mean p-value of log-rank test with different values of b. Dashed blue lines represents the 95% CI. Red dashed line represents the statistical significance level p = 0.05. Simulations were repeated 100 times. C) Effect in α D) Effect in μ. (PDF) [file pcbi.1010444.s002.pdf]

S2 Fig: Discrete Covariate

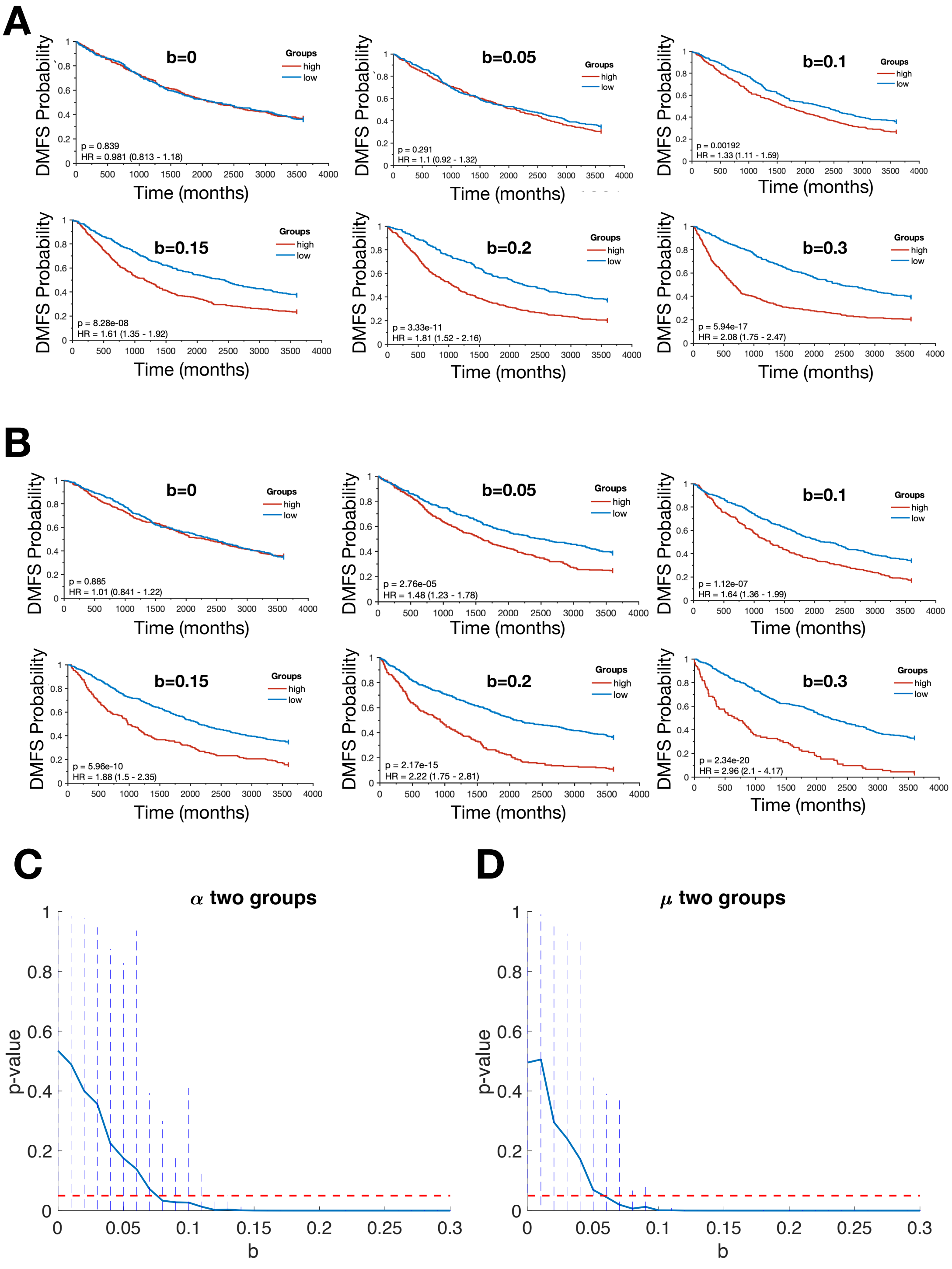

Survival curves between groups with different levels values of b: A) Effect in α, B) effect in μ.
C-D)Solid line represents the mean p-value of log-rank test with different values of b. Dashed blue lines represents the 95% CI. Red dashed line represents the statistical significance level $p = 0.05$. Simulations were repeated 100 times. C) Effect in α D) Effect in μ
