# Supplementary material for: Practical identifiability analysis of a mechanistic model for the time to distant metastatic relapse and its application to renal cell carcinoma
Source: PLoS Comput Biol. 2022 Aug 25;18(8):e1010444. doi: 10.1371/journal.pcbi.1010444 (PMC9451098; doi:10.1371/journal.pcbi.1010444)
Supplement: S3 Fig — Synthetic DMFS curves at different thresholds simulated with a effect in the variable α. P-values at different thresholds are displayed in the center figure. A) Group effect B) Linear effect. (PDF) [file pcbi.1010444.s003.pdf]

S3 Fig: Different effects in a continuos variable

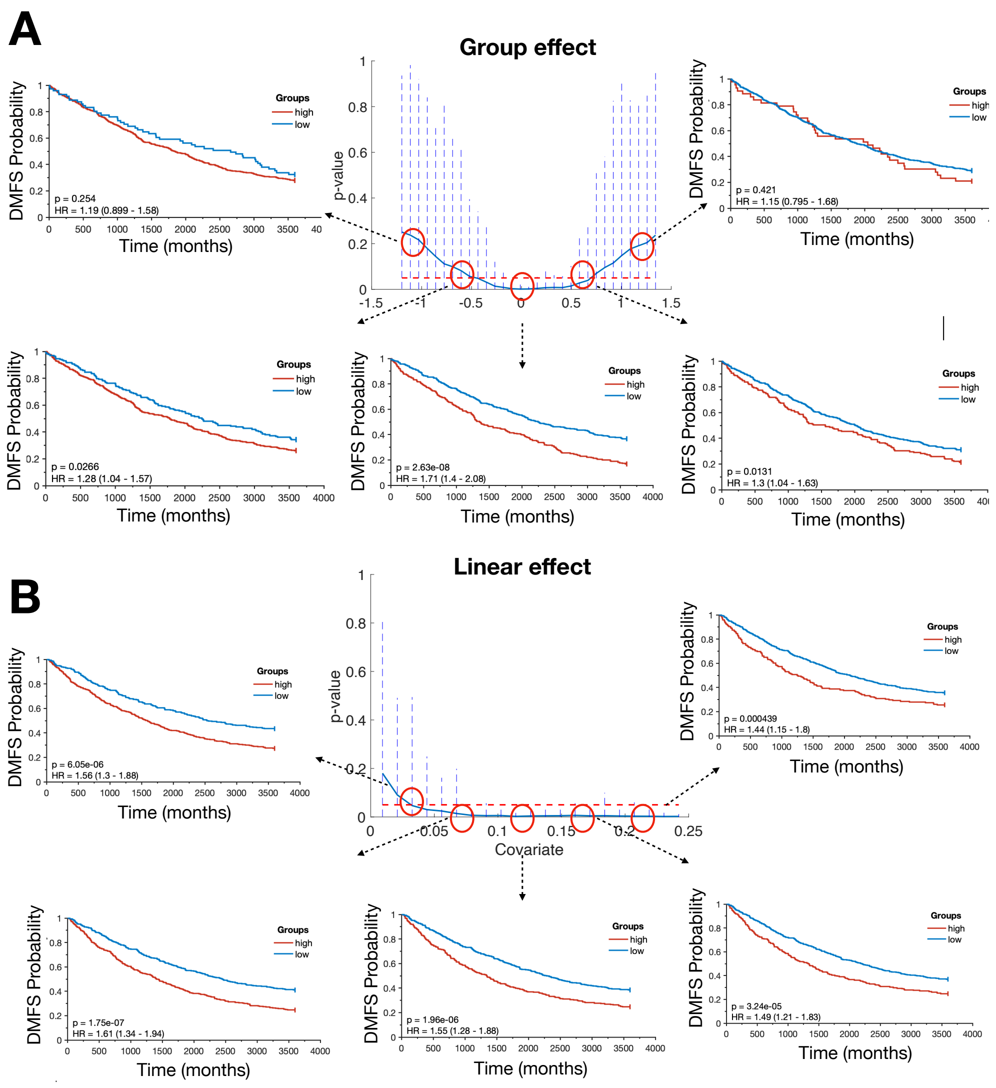

Synthetic DMFS curves at different thresholds simulated with a effect in the variable α. P-values at different thresholds are displayed in the center figure. A) Group effect B) Linear effect
