# Supplementary material for: Practical identifiability analysis of a mechanistic model for the time to distant metastatic relapse and its application to renal cell carcinoma
Source: PLoS Comput Biol. 2022 Aug 25;18(8):e1010444. doi: 10.1371/journal.pcbi.1010444 (PMC9451098; doi:10.1371/journal.pcbi.1010444)
Supplement: S4 Fig — Results from minimization process. Number 1–3 refers to effects group, linear, group and linear in the variable αpop and 4–6 in the variable μpop. Red boxplots correspond to the real model used to generate the data with the model. A) Normal distribution for the covariate, B) Lognormal distribution. (PDF) [file pcbi.1010444.s004.pdf]

S4 Fig: Identification of the right effect with a continuous covariate

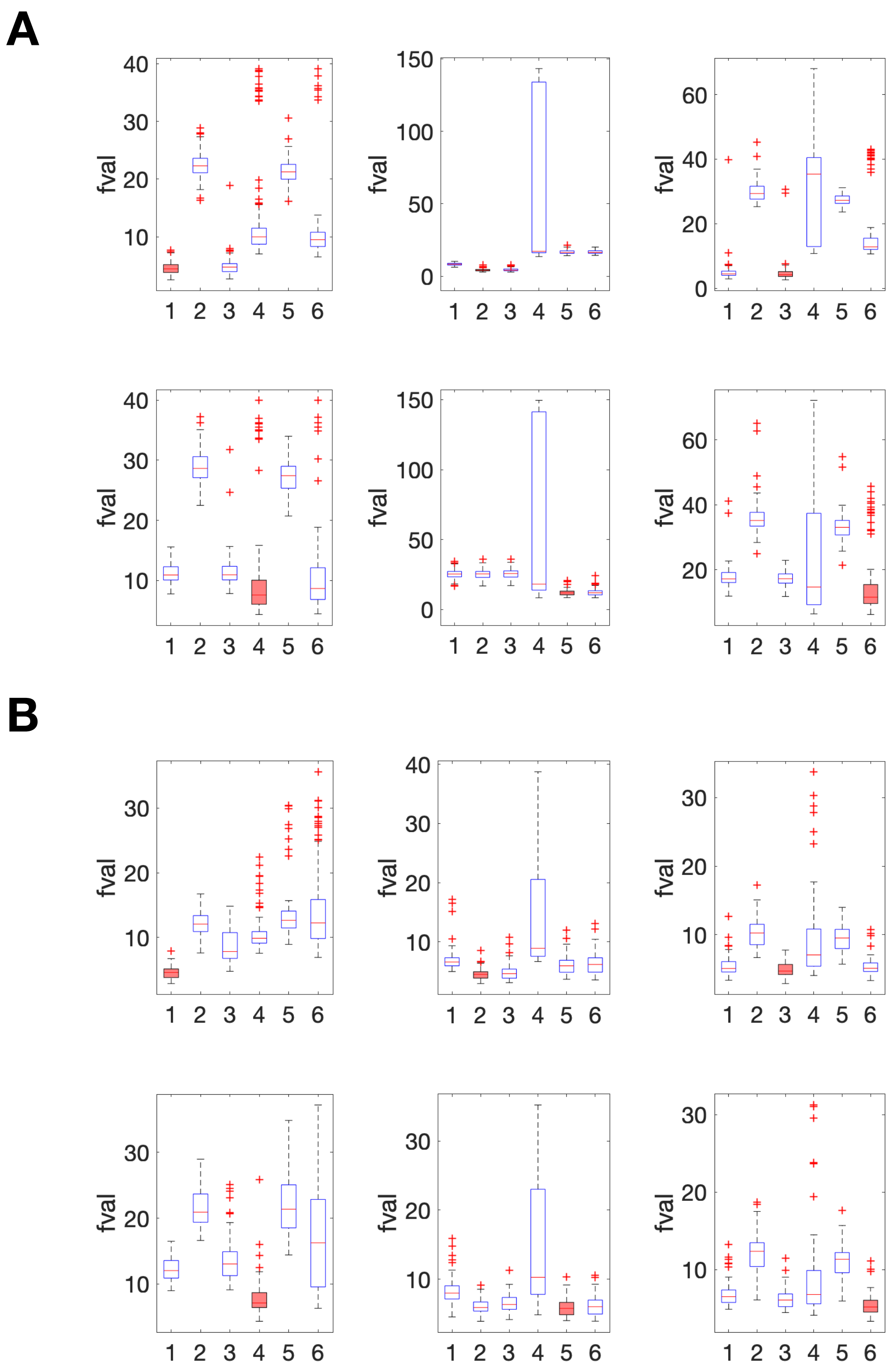

Results from minimization process. Number 1-3 refers to effects group, linear, group and linear in the variable α_{pop} and 4-6 in the variable μ_{pop}. Red boxplots correspond to the real model used to generate the data with the model. A) Normal distribution for the covariate, B) Lognormal distribution.
